# Efficient privacy-preserving string search and an application in genomics

**DOI:** 10.1093/bioinformatics/btw050

**Published:** 2016-03-02

**Authors:** Kana Shimizu, Koji Nuida, Gunnar Rätsch

**Affiliations:** 1 Biotechnology Research Institute for Drug Discovery, National Institute of Advanced Industrial Science and Technology, Tokyo 135-0064, Japan,; 2 Computational Biology, Memorial Sloan Kettering Cancer Center, New York, NY 1275 York, USA,; 3 Information Technology Research Institute, National Institute of Advanced Industrial Science and Technology, Tokyo 135-0064, Japan and; 4 Japan Science and Technology Agency (JST) PRESTO Researcher, Tokyo, Japan

## Abstract

**Motivation**: Personal genomes carry inherent privacy risks and protecting privacy poses major social and technological challenges. We consider the case where a user searches for genetic information (e.g. an allele) on a server that stores a large genomic database and aims to receive allele-associated information. The user would like to keep the query and result private and the server the database.

**Approach**: We propose a novel approach that combines efficient string data structures such as the *Burrows–Wheeler transform* with cryptographic techniques based on additive homomorphic encryption. We assume that the sequence data is searchable in efficient iterative query operations over a large indexed dictionary, for instance, from large genome collections and employing the (positional) Burrows–Wheeler transform. We use a technique called *oblivious transfer* that is based on *additive homomorphic encryption* to conceal the sequence query and the genomic region of interest in positional queries.

**Results**: We designed and implemented an efficient algorithm for searching sequences of SNPs in large genome databases. During search, the user can only identify the longest match while the server does not learn which sequence of SNPs the user queried. In an experiment based on 2184 aligned haploid genomes from the 1000 Genomes Project, our algorithm was able to perform typical queries within ≈ 4.6 s and ≈ 10.8 s for client and server side, respectively, on laptop computers. The presented algorithm is at least one order of magnitude faster than an exhaustive baseline algorithm.

**Availability and implementation:** https://github.com/iskana/PBWT-sec and https://github.com/ratschlab/PBWT-sec.

**Contacts:** shimizu-kana@aist.go.jp or Gunnar.Ratsch@ratschlab.org

**Supplementary information:** Supplementary data are available at *Bioinformatics* online.

## 1 Introduction

String search is a fundamental task in the field of genome informatics, for which a large variety of techniques have been developed (see, for instance, Altschul *et al.*, 1990; Kent, 2002; Li and Homer, 2010). Traditionally, those techniques have been optimized for accuracy and computational efficiency, however a recent boom of personal genome sequencing and analyses has spotlighted a new criteria, namely, privacy protection. As reported in many studies, a genome is considered to be one of the most critical pieces of information for an individual’s privacy. In fact, it is largely different from any other personal information because it works as an identifier of an individual while it possesses the information that has strong correlation with the phenotype of the individual (Erlich and Narayanan, 2014; Roche and Annas, 2001). Therefore, in principle, privacy protection is an inevitable problem when handling personal genomes. As a practice, the most popular approach is protecting genomes physically; genomic sequences have been kept at few collaborator sites, and only a limited number of researchers are allowed to access them. This conservative approach severely limits the great potential of existing genomic resources. In order to mitigate the stagnation caused by privacy issues, it appears crucial to develop practical methods that enable searching and mining genomic databases in a privacy-preserving manner.

So far, several groups have tackled related problems. Jha *et al.* (2008) developed secure multi-party computation protocols for computing edit distance. Blanton and Aliasgari (2010) proposed a protocol to search DNA string against a DNA profile represented by finite automata. Bruekers *et al.* (2008) proposed a protocol to detect a match between two short DNA sequences for the purpose of genetic test. Baldi *et al.* (2011) also aimed for genetic test to develop a method for computing set intersection cardinality. Freedman *et al.* (2005) proposed a protocol for searching pre-defined keywords from databases. Naganuma *et al.* (2012) proposed a substring search protocol for public databases while keeping user’s query private. Ayday *et al.* (2013) developed a system by using several cryptographic techniques to find a subset of short reads which includes a fixed-length query string at specific position. He *et al.* (2014) proposed an algorithm for finding relatives by secure identity-by-descent matches.

We propose a general approach which utilizes an efficient iteratively queriable data structure together with cryptographic techniques. Among many variations of such data structures, the Burrows–Wheeler Transform (BWT Langmead *et al.*, 2009; Li and Durbin, 2009; Li *et al.*, 2009) and related techniques such as the positional BWT (PBWT; Durbin, 2014) have dramatically improved the speed of genomic database analyses. Those data structures commonly have an indexed dictionary called a rank dictionary. By referring to the rank dictionary in iterative operations, one can efficiently search the database. For the case of BWT, a match between query and database is reported as a left-open, right-closed interval (f,g], and the interval is computed by the look-up of the rank dictionary. In our approach, we access the rank dictionary in privacy-preserving manner by using *additive homomorphic encryption* and *oblivious transfer* (OT).

Cryptographic approaches often require significant computational resources. The goal of this work is to illustrate that privacy-preserving queries are within reach when using current cryptographic techniques and standard computing hardware. We demonstrate that a typical query would only take about 4.6 s on the user side using a single thread and ≈ 10.8 s on the server having four cores, while preserving privacy of the query string and the database.

The rest of the paper is organized as follows. In Approach, we describe the main ideas of our approach without going into technical details. In Methods, the detailed algorithm of recursive oblivious transfer is given followed by the description of a practical algorithm, named PBWT-sec, for privacy-preserving search in large-scale genotype databases. We also describe complexity and security properties of the proposed algorithm. We provide the more intricate details of a more efficient version of the algorithm in Supplementary Sections S1–S2. In Experiments, we evaluate the performance of PBWT-sec on datasets created from data of the 1000 Genomes Project (The 1000 Genomes Project Consortium, 2012) and compare it to an alternative method for fixed-length *k*-mer search. Finally, we conclude our study in Section 5.

## 2 Approach

### 2.1 Problem setup

We consider the setting in which a user would like to search a genomic sequence in a database with the aim to either determine whether this sequence exists in the queried database and/or to obtain additional information associated with the genomic sequence. An example is the use in a so-called *genomic beacon* (for instance, those created within the *Beacon Project* of the Global Alliance for Genome & Health (GA4GH).) Another application is the search of a specific combination of variants, for instance, in the BRCA1 or BRCA2 genes, with the aim to determine whether that combination of variants is known or predicted to be deleterious (see, for instance, GA4GH’s *BRCA Challenge*). For privacy reasons, the user would like to conceal the queried sequence, which would be particularly relevant for the second example. For both examples it would be important that the server’s database is protected.

### 2.2 Information flow of searches on recursive search data structures

Let us describe the information flow between a user and a server for such problems. In this work, we perform searches on the (positional) Burrows–Wheeler transform of a genomic database of length *N*. (P)BWT stores string information very efficiently and still allows computations (this is a property of Succinct Data Structures, see Jacobson, 1988).

To search for a query string *q* over the alphabet Σ, one iteratively operates on intervals that can later be used to identify the matching genomic regions based on the (P)BWT. A substring match is represented by an interval (f,g]. The number of matches is given by the length of the interval *g*–*f*. It is known that the (k+1)th interval $\left(f_{k+1},g_{k+1}\right]$ corresponding to a (k+1)-mer match can be updated from the *k*th interval $\left(f_{k},g_{k}\right]$ and the (k+1)th letter of the query *q*.

We will provide more details on how to update *f* and *g* in Section 3.3. To understand the key ideas, it is sufficient to understand that the updates can be written in the form of

$$f_{k+1}=v_{c}[f_{k}]\text{ and }g_{k+1}=v_{c}[g_{k}],$$

where c=q[k+1] and $v_{c}\in N^{N}$ is a large, static lookup table. Hence, the iterative algorithm of updating $\left(f_{k},g_{k}\right]$ by using the query *q*, can be written as a recursive algorithm:

$$f_{k+1}=v_{q[k+1]}[v_{q[k]}[v_{q[k-1]}[\ldots v_{q[1]}[f_{0}]\ldots ]]].$$

**Fig. 1.**
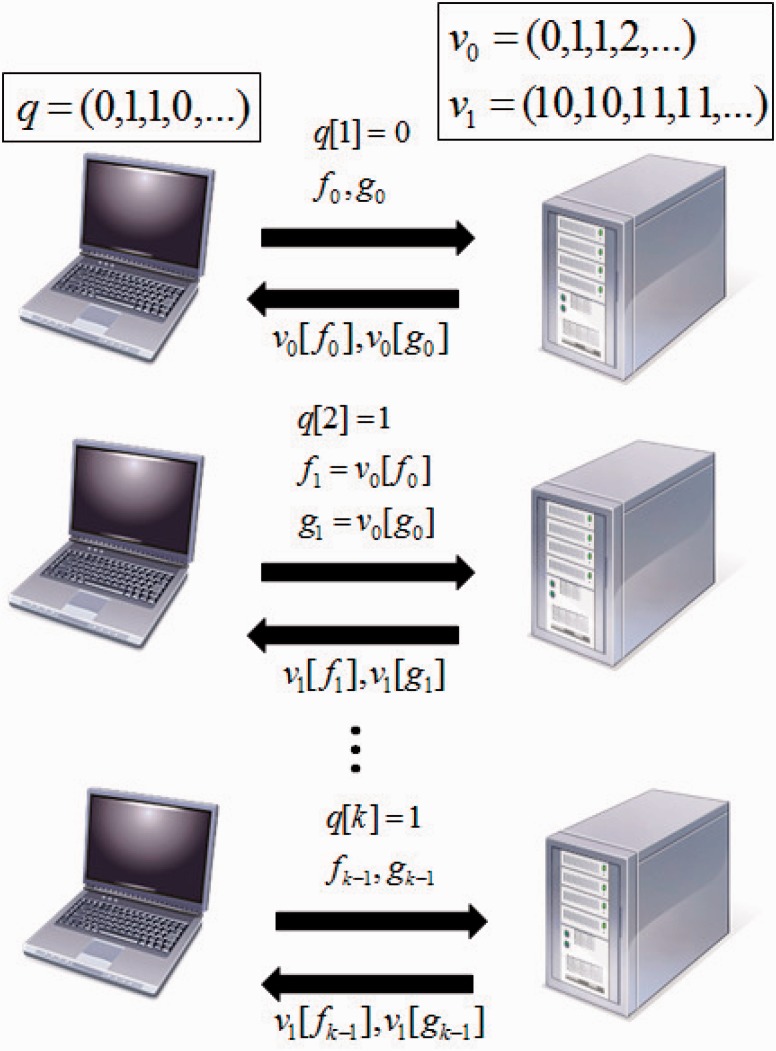
Information flow of a search on a recursive search data structure such as (P)BWT. For *i*th iteration, the user sends $q[i],f_{i-1}$, and $g_{i-1}$, the server returns $v_{q[i]}[f_{i-1}]$ and $v_{q[i]}[g_{i-1}]$, and the user updates $f_{i}=v_{q[i]}[f_{i-1}]$ and $g_{i}=v_{q[i]}[g_{i-1}]$

This can be done analogously for $g_{k+1}$. In this work we will refer to data structures that can be queried in the recursive way described above as *recursive search data structures*. Figure 1 illustrates the information flow of a search on the recursive search data structure.

### 2.3 Oblivious transfer for privacy-preserving search

In a search on the recursive search data structures, the user needs to conceal not only a query string *q* but also *f* and *g* because *f_i_* is $f_{i-1}$th element of $v_{q[i]}$, and q[i] is inferred from those two values. Analogously, q[i] is also inferred from *g_i_* and $g_{i-1}$. The server needs to minimize output because the user reconstructs a part of v from the server’s output. In this study, we achieve such security requirements by a cryptographic technique called *oblivious transfer*.

#### 2.3.1 Oblivious transfer

**Fig. 2.**
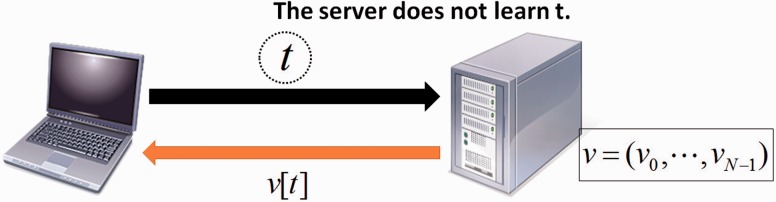
Outline of Oblivious Transfer (OT). The user (laptop computer) obtains *t*th element of the server’s vector v without leaking *t* to the server, and none of the other elements of the vector v

*Oblivious transfer* (OT) is a cryptographic technique for two parties: the user and the server, and enables the user to specify 0≤t<N and obtain only *t*th element of the server’s vector v without leaking any information about *t* to the server (Rabin, 1981). Figure 2 illustrates an outline of the oblivious transfer. Among several efficient algorithms (Lipmaa, 2005, 2008; Zhang *et al.*, 2013), we used those which are based on additive homomorphic encryption. The detailed algorithm will be given in Section 3.2.

#### 2.3.2 Concealing the query

The user’s query consists of $\left(f_{i},\;g_{i}\right]$, and q[i+1] for *i*th iteration. A key idea of our approach is to look-up elements of $v_{c}$ by OT and obtain the next interval $\left(f_{i+1}=v_{c}[f_{i}],\;g_{i+1}=v_{c}[g_{i}]\right]$ without revealing $\left(f_{i},\;g_{i}\right]$ to the server. In our approach, we also use a masking technique such that the user tries $v_{c}[f_{i}]$ for all c∈Σ, and the server only returns $v_{c}[f_{i}]$ where c=q[i+1] without knowing the value of q[i+1]. Technical details will be given in Section 3.2.

#### 2.3.3 Concealing the database

While this approach protects a user’s privacy, the server leaks information of $v_{c}$ which may be sufficient to reconstruct parts of the genotypes in the database. In order to rigorously protect the server’s privacy, we propose a technique that allows for recursive oblivious transfer where the user does not learn intermediate results but only if a unique match was found. It is based on a bit-rotation technique which enables the server to return ${\hat{f}}_{k}:=R(f_{k})$ and ${\hat{g}}_{k}:=R\prime (g_{k})$ which are random values to the user. Only the server can recover *f_k_* and *g_k_ in encrypted form* (i.e. the server does not see *f_k_* and *g_k_* when recovering them), and thus the user can recursively access $v_{c}[f_{k}]$ and $v_{c}[g_{k}]$ correctly. The details of this approach are given in Section 3.2.

In this work, we designed an algorithm based on these techniques that can be used for privacy-preserving search in large genotype databases.

Note that there are still privacy risks for the server, though returning only a unique match minimizes the information leakage from the server. For example, assume there is a database storing a genomic study of drug addicts that implements the PBWT-sec, and a person (Bob) participated in the study. If someone obtains a DNA sample from Bob and queries the databases, he/she will reveal that Bob is a drug addict. As described in the above case, there is always a limitation for protecting the server’s privacy as long as the server returns the search results, and there is associated information such as phenotypes (Shringarpure and Bustamante, 2015). We emphasize that this issue is common for any database search application and is not specific to our proposed method.

## 3 Methods

### 3.1 Additively homomorphic encryption

Our main cryptographic tool in this paper is an additive-homomorphic public-key encryption scheme (KeyGen;Enc;Dec), which enables us to perform additive operations on *encrypted* values. Here, the algorithm KeyGen generates a public key pk and a secret key sk; Enc(m) denotes a ciphertext obtained by encrypting message *m* under the given pk; and Dec(c) denotes the decryption result of ciphertext *c* under the given sk. The scheme also has the following additive-homomorphic properties:

- Given two ciphertexts $\mathrm{Enc}(m_{1})$ and $\mathrm{Enc}(m_{2})$ of integer messages *m*_1_ and *m*_2_, $\mathrm{Enc}(m_{1}+m_{2})$ can be computed without knowing *m*_1_, *m*_2_ and the secret key (denoted by $\mathrm{Enc}(m_{1})⊕\mathrm{Enc}(m_{2})$).
- Given a ciphertext Enc(m) of a message *m* and an integer *e*, Enc(e·m) can be computed without knowing *m* and the secret key (denoted by e⊗Enc(m)). In particular, Enc(−m) can be computed in this manner.

This scheme should have semantic security; that is, a cipher text leaks no information about the original message (Goldwasser and Micali, 1984). For example, we can use either the Paillier cryptosystem (Paillier, 1999) or the ‘lifted’ version of the ElGamal cryptosystem (ElGamal, 1985); now the second operation ⊗ can be realized by repeating the first operation ⊕.

Algorithm 1. Recursive oblivious transfer

1: **function** PrepQuery(*t*, *N*)

2:   $q=(q_{0}=0,\ldots ,q_{t}=1,\ldots ,q_{N-1}=0)$

3:   $\vec{\mathrm{Enc}}(q)=(\mathrm{Enc}(q_{0}),\ldots ,\mathrm{Enc}(q_{N-1}))$

4:   **return** $\vec{\mathrm{Enc}}(q)$

5: **end function**

6:

7: **function** ROT($\vec{\mathrm{Enc}}(\hat{q}),\;v$, *r*, r′, *N*)

8:   $\vec{\mathrm{Enc}}(q\prime )=\mathrm{Perm}(\vec{\mathrm{Enc}}(\hat{q}),r\prime )$

9:    $\hat{c}={⊕}_{i=0}^{N-1}(({\left(v[i]+r\right)}_{\text{mod }N}⊗\mathrm{Enc}(q\prime_{i}))$

10:   **return** $\hat{c}$

11: **end function**

12:

13: v is a server’s private vector of length *N*.

14: *x*_1_ is a user’s private value.

15: $x_{\ell }$ is the value of user’s interest.

16: ℓ is known to both user and server.

17: User’s initialization: $t\leftarrow x_{1}$

18: Server’s initialization: r′←0

19: Common initialization: i←1

20: **while** i<ℓ **do**

21:   The user computes: $\vec{\mathrm{Enc}}(q)\leftarrow \mathrm{PrepQuery}(t,N)$

22:   **if** i==(ℓ−1) **then**

23:    Server sets: *r* = 0

24:   **else**

25:    Server generates random value *r*

26:   **end if**

27:   Server computes: $\hat{c}\leftarrow$ ROT($\vec{\mathrm{Enc}}(q),\;v$, *r*, r′, *N*)

28:   Server sets: r′←r

29:   Server sends $\hat{c}$ to user

30:   User computes: $t\leftarrow \mathrm{Dec}(\hat{c})$

31: **end while**

32: User obtains $x_{\ell }=t$.

**Fig. 3.**
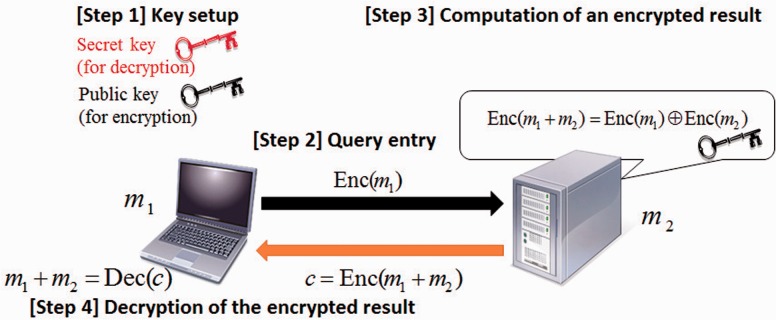
Computation of $m_{1}+m_{2}$ on the server in encrypted form by additively homomorphic encryption

Figure 3 illustrates an outline of performing an additive operation on a user’s value *m*_1_ and a server’s value *m*_2_ by the additively homomorphic encryption. In the first step, the user generates two keys: a secret key and a public key, and the user sends the public key to the server. In the second step, the user encrypts *m*_1_ by the public key and sends a ciphertext $\mathrm{Enc}(m_{1})$ to the server. In the third step, the server encrypts *m*_2_ by the public key and computes $c=\mathrm{Enc}(m_{1}+m_{2})$. The server sends a ciphertext *c* to the user. In the fourth step, the user obtains $m_{1}+m_{2}$ by decrypting *c*.

It goes beyond the scope of this paper to review the details of these cryptographic techniques and the reader is referred to a book (Yi *et al.*, 2014) on homomorphic encryption. A typical addition operation in the ElGamal cryptosystem takes about $2\times 10^{-7}\text{ s}$ on a single CPU based on AIST’s ElGamal encryption library (https://github.com/aistcrypt/Lifted-ElGamal).

### 3.2 Recursive oblivious transfer by random rotations

To protect the privacy of the database, we propose a technique for recursively querying a data structure without obtaining information about intermediate results. Let us define the recursive oblivious transfer problem as follows:

Model 1. *A user has a private value* $0\leq x_{1}<N$ *and a server has a private vector* v *of length N. Let us denote* $x_{k+1}=v[x_{k}]$ *and the user is allowed to access the server* ℓ−1 *times. After the calculation, the user learns only* $x_{\ell }$ *and the server learns nothing about* $x_{1},\ldots ,x_{\ell }$.

Here we explain our idea by extending a simple linear communication size OT where the user aims to know the *t*th element of the server’s vector v.

**Fig. 4.**
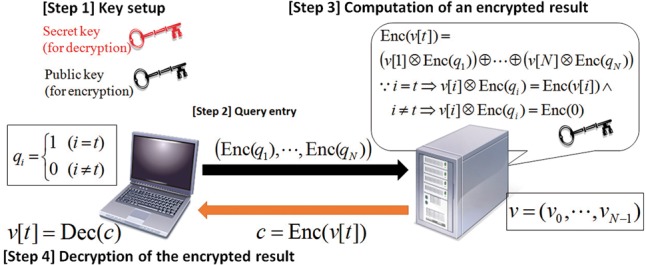
Outline of Oblivious Transfer (OT) based on additive homomorphic encryption. See Sections 2.3 and 3.2 for more details

Figure 4 illustrates the oblivious transfer algorithm based on additive homomorphic encryption. In the initialization step, the user generates a public key and a secret key and sends the public key to the server. The user creates a bit vector:

$$q=(q_{0}=0,\ldots ,q_{t}=1,\ldots ,q_{N-1}=0)\;,$$

and sends the following encrypted vector to the server.

$$\vec{\mathrm{Enc}}(q)=(\mathrm{Enc}(q_{0})\ldots ,\mathrm{Enc}(q_{N-1}))$$

The server computes

$$c={⊕}_{i=0}^{N-1}(v[i]⊗\mathrm{Enc}(q_{i})),$$

and sends *c* to the user.

The user computes Dec(c) and obtains v[t] by using the secret key because $i=t\Rightarrow v[t]⊗\mathrm{Enc}(q_{t})=\mathrm{Enc}(v[i])$ and $i\neq t\Rightarrow v[t]⊗\mathrm{Enc}(q_{t})=\mathrm{Enc}(0)$.

Now we consider the case that the server does not leak v[t], but allows the user to access v[v[t]]. Our idea is that the server generates a random value r∈{0,1,…,N−1} and returns the cipher text

$$\hat{c}={⊕}_{i=0}^{N-1}(({\left(v[i]+r\right)}_{\text{mod}\;\;N})⊗\mathrm{Enc}(q_{i}))=\mathrm{Enc}({\left(v[t]+r\right)}_{\text{mod}\;\;N}),$$

where ${\left(a+b\right)}_{\text{mod}\;\;N}$ denotes addition in a number field modulo *N*. The user decrypts $\hat{c}$ to know a randomized result ${\left(v[t]+r\right)}_{\text{mod}\;\;N}$, and performs the next query:

$$\hat{q}=({\hat{q}}_{0}=0,\ldots ,{\hat{q}}_{\left({\left(v[t]+r\right)}_{\text{mod}\;\;N}\right)}=1,\ldots ,{\hat{q}}_{N-1}=0).$$

Note that $\hat{q}$ is the *r*-rotated permutation of the ‘true’ query:

$$q\prime =(q\prime_{0}=0,\ldots ,q\prime_{v[t]}=1,\ldots ,q\prime_{N-1}=0).$$

Therefore, denote Perm(q,r) as the permutation of a vector q such that *i*th element moves to $\left({\left(i-r\right)}_{\text{mod }N})\right)$th position, the server can correctly recover ‘true’ query q′ in its encrypted form by the following permutation: $\vec{\mathrm{Enc}}(q\prime )=\mathrm{Perm}(\vec{\mathrm{Enc}}(\hat{q}),r)$. In this way, the server correctly computes an encrypted v[t]th element by

$$\mathrm{Enc}(v[v[t]])={⊕}_{i=0}^{N-1}(v[i]⊗\mathrm{Enc}(q\prime_{i})),$$

without learning any information about the user’s query.

By recursively applying these calculations, the user can obtain $x_{k+1}$ according to Model 1. The complete algorithm implementing this idea is given in Algorithm 1. It uses a function ROT for rotating the server’s results to conceal intermediate query results in order to protect the database.

Algorithm 2. . The detailed description of PBWT-sec finding a set-longest match at position *t*.

• Public input: Problem size *M* & *N*; alphabet Σ={0,1,..,|Σ|−1}, start position t∈{1,…,N}

• Private input of user: A query sequence *S* of length ℓ

• Private input of server: PBWT matrix $P\in N^{M\times N}$

0. (*Key setup of cryptosystem*) User generates key pair (pk,sk) by key generation algorithm KeyGen for additive-homomorphic cryptosystem and sends public key pk to server.

1. (*User initialization*) Set initial interval (f=0,g=M].

2. (*Recursive search*)

Initializes query and position index: i←1; k←t−1

**while** (i≤ℓ) **do**

a. (*Query entry*) The user performs the following steps:

 • Prepares next query:

      $\vec{\mathrm{Enc}}(q_{f})\leftarrow$ PrepQuery(f,M+1)

      $\vec{\mathrm{Enc}}(q_{g})\leftarrow$ PrepQuery(g,M+1)

    • Sends $\mathrm{Enc}(S[i]),\;\vec{\mathrm{Enc}}(q_{f}),\;\vec{\mathrm{Enc}}(q_{g})$ to the server.

b. (*Search*) The server performs the following steps:

    • Compute look-up tables for all c∈Σ:

$v_{c}[j]=\;\{\begin{array}{ll} CF_{c}(P_{\cdot ,k}) & \left(j=0\right)\\ CF_{c}(P_{\cdot ,k})+\mathrm{Ran}k_{c}(P_{\cdot ,k},j) & \left(1\leq j\leq M\right) \end{array}\;$

    • Obtain random values $r^{\left(f\right)}$, $r^{\left(g\right)}$

    • Set $r\prime^{\left(f\right)}=r\prime^{\left(g\right)}=0$ iff. i==0

    • Compute next possible intervals for all c∈Σ:

     $e_{c}^{\left(f\right)}$ ← $\mathrm{ROT}(\vec{\mathrm{Enc}}(q_{f}),v_{c},r^{\left(f\right)},r\prime^{\left(f\right)},M)$

     $e_{c}^{\left(g\right)}$ ← $\mathrm{ROT}(\vec{\mathrm{Enc}}(q_{g}),v_{c},r^{\left(g\right)},r\prime^{\left(g\right)},M)$

    • Randomize return values except for user’s target interval by computing the following for all c∈Σ

    Generate temporary random values *r*_0_, *r*_1_

     $e_{c}^{\left(f\right)}$ ← $e_{c}^{\left(f\right)}$ ⊕ $\mathrm{Enc}(r_{0}\times (S[i]-c))$

     $e_{c}^{\left(g\right)}$ ← $e_{c}^{\left(g\right)}$ ⊕ $\mathrm{Enc}(r_{1}\times (S[i]-c))$

    • Compute an encrypted flag showing if match is longest

d← isLongest $\left(\vec{\mathrm{Enc}}(q_{f}\right)$, $\vec{\mathrm{Enc}}(q_{g})$, $r\prime^{\left(f\right)}$, $r\prime^{\left(g\right)})$

    • Store random values $r\prime^{\left(f\right)}\leftarrow r^{\left(f\right)}$, $r\prime^{\left(g\right)}\leftarrow r^{\left(g\right)}$

    • Send *d*, $e^{\left(f\right)},\;e^{\left(g\right)}$ to the user

c. (*Decryption of encrypted flag and randomized interval*)

The user performs the following steps:

 **if** (Dec(d)==0)

  Sends decoy queries to server until i==ℓ

  Reports result S[1,…,i−2]

 **else**

  Computes $f\leftarrow \mathrm{Dec}(e_{S[i]}^{\left(f\right)})$, $g\leftarrow \mathrm{Dec}(e_{S[i]}^{\left(g\right)})$,

 **end if**

i←i+1, k←k+1

**end while**

### 3.3 *PBWT-sec*: Privacy-preserving search on genotype databases

In this section, we introduce a practical genotype database search based on recursive oblivious transfer and PBWT. We only introduce the algorithm to search for the longest match starting from *t*th column, however, variations are possible and would allow for a variety of different search types (see also Durbin, 2014).

To formulate the problem, let us consider a set *X* of *M* haplotype sequences *x_i_*, i=1,…,M over *N* genomic positions indexed by k=1,…,N, and a query *q* which is a user’s haplotype sequence over the same *N* genomic positions. We denote *k*th allele of a sequence *x_i_* by $x_{i}[k]$. Given two indices *k*_1_ and *k*_2_, we say that there is a match between *q* and *x_i_* from *k*_1_ to *k*_2_, if $q[k_{1}]\ldots q[k_{2}-1]=x_{i}[k_{1}]\ldots x_{i}[k_{2}-1]$. We say that the match is set-longest at *k*_1_ if there is no match between *q* and any sequence *x_j_* (possibly with *j *=* i*) from *k*_1_ to $k_{2}+1$.

**Fig. 5.**
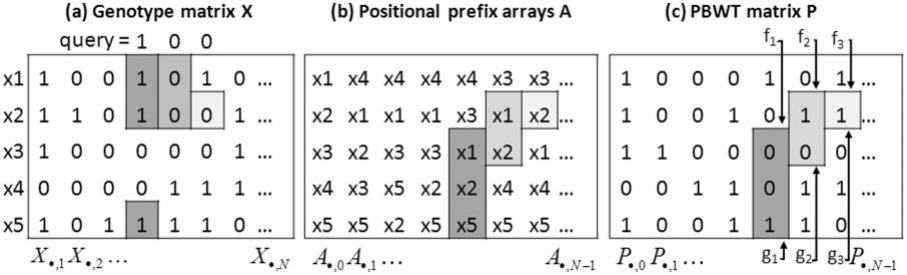
Outline of the search strategy with PBWT. A set of genotype sequences $X=\{x_{1},\ldots ,x_{5}\}$ illustrated in (**a**) is sorted by the algorithm described in Durbin (2014) to obtain the positional prefix arrays *A* illustrated in (**b**). Each element $P_{i,j}$ of PBWT matrix illustrated in (**c**) is (j+1)th letter of sequence $A_{i,j}$. By computing rank operations with regard to *k*th query letter on $P_{\cdot ,k-1}$, one can update an interval corresponding to *k*-mer match between the query and *X*. In this figure, the search starts from fourth allele. The first interval $\left(f_{1},g_{1}\right]$ is initialized by rank operations on $P_{\cdot ,3}$ with regard to first query letter ‘1’. The second interval $\left(f_{2},g_{2}\right]$ is obtained by rank operations on $P_{\cdot ,4}$ with regard to the second query letter ‘0’ and $\left(f_{1},g_{1}\right]$. Similarly, the third interval $\left(f_{3},g_{3}\right]$ is obtained by rank operations on $P_{\cdot ,5}$ with regard to the third query letter ‘0’ and $\left(f_{2},g_{2}\right]$. See Sections 2.2 and 3.3 for more details

The goal is to find a set-longest match at a given position *t* between *q* and *X* in a privacy-preserving manner. Here, we consider the case that the user’s private information is the query string and the position *t* is not the user’s private information. We later introduce the case that the both the query string and *t* are user’s private information. The formal description of the model is described as follows:

Model 2. *The user is a private haplotype sequence holder, and the server is a holder of a set of private haplotype sequences. The user learns nothing but a set-longest match at a given position t between the query and the database while the server learns nothing about the user’s query. t is not a user’s private information and the server knows it*.

Let us remember how to search the set-longest match in *non*-privacy-preserving manner. PBWT involves a matrix $P\in N^{M\times N}$ that stores well-compressible information in an efficiently searchable form. It is created from the genotype matrix *X* by algorithms described in Durbin (2014) such that *i*th column is (i+1)th letters of sequences sorted by *i* reverse prefix (i.e. sorted from *i*th letter to first letter). In order to compute the match starting from the first allele, *P* has 0th column $P_{\cdot ,0}={\left(x_{1}[1],\ldots ,x_{M}[1]\right)}^{T}$. By using rank dictionary operations on *P* (see below), one can search a match between a query and *X*. When operating on *P* one computes updates of intervals using the following two quantities (see Durbin, 2014 for more details): (i) The rank dictionary for sequence *S* for letter c∈Σ at position *t*:

$$\mathrm{Ran}k_{c}(S,t)=|\{\;j\;|\;S[j]=c\;,\;1\leq j\leq t\;\}|\;,$$

where Σ is the alphabet of *S*. (ii) The table CF counting occurrences of letters that are lexicographically smaller than *c* in *S* by

$$CF_{c}(S)=\sum_{r<c}\mathrm{Ran}k_{r}(S,N)\;.$$

Based on these two quantities, we can compute the updates $\left(f_{k+1},g_{k+1}\right]$ using two simple operations

$$f_{k+1}=CF_{c}(P_{\cdot ,k})+\mathrm{Ran}k_{c}(P_{\cdot ,k},f_{k})\;,$$

$$g_{k+1}=CF_{c}(P_{\cdot ,k})+\mathrm{Ran}k_{c}(P_{\cdot ,k},g_{k})\;,$$

where we denoted the *k*th column vector by $P_{\cdot ,k}$. Let us define a look-up vector $v_{c}$ for the column *k* where

(1) $$v_{c}[i]=\{\begin{array}{ll} CF_{c}(P_{\cdot ,k}) & \left(i=0\right)\\ CF_{c}(P_{\cdot ,k})+\mathrm{Ran}k_{c}(P_{\cdot ,k},i) & \left(1\leq i\leq M\right) \end{array}\;$$

for c∈Σ. Then, updating an interval is equivalent to two look-ups in the vector $v_{c}$:

(2) $$f_{k+1}=v_{c}[f_{k}]\text{ and }g_{k+1}=v_{c}[g_{k}].$$

Given a position *t* and a PBWT *P* of the database sequences, the first match is obtained as an interval $\left(f_{1}=v_{c}[0],g_{1}=v_{c}[M]\right]$ where c=q[1] and $v_{c}$ is a look-up vector for (t−1)th column of *P* (see the definition of $v_{c}$ in Equation (1)). The match is extended by one letter by an update of the interval. The update from the *k*th interval to (k+1)th interval is conducted by specifying c=q[k+1], re-computing *v_c_* for (k+1)th column of *P* and referring $v_{c}[f_{k}]$ and $v_{c}[g_{k}]$ as $f_{k+1}$ and $g_{k+1}$ (see Equation (2)). The set-longest-match is found when *f *=* g*. An outline of the search using PBWT is illustrated in Figure 5.

In order to achieve the security described in the model 2, for each update, the user has to specify *c* without leaking *c* to the server, and obtain only $v_{c}[f]$ and $v_{c}[g]$ without leaking *f* and *g*. To satisfy the second requirement, the user accesses the server’s $v_{c}$ through the function ROT, which allows the user to obtain a specific element in the specified vector. To achieve the first requirement, the server computes all possible intervals (i.e. computing (*f*, *g*] for the all case of c=0,…,|Σ|−1). This allows the user to obtain the correct interval, however, the sever leaks extra information (i.e. intervals for c≠q[k]). To avoid this, the user sends Enc(q[k]), and the server adds a conditional randomization factor r×(q[k]−c) to *f* and *g* with different random value *r* for all c∈Σ. Note that this factor becomes equivalent to 0 iff. q[k]=c, and user only obtains the interval for c=q[k].

In order to identify the set-longest match, the user has to know if *f *=* g*. The user cannot compute the identity of *f* and *g* directly from the server’s return, because ROT returns a value which is a random value to the user (but the ‘true’ value is recovered in encrypted form only at the server side). Therefore, the server also sends an encrypted flag *d* which shows whether or not *f *=* g*. Since *f* and *g* are represented as indices of $q\prime_{f}=\mathrm{Perm}(q_{f},r\prime^{\left(f\right)})$ and $q\prime_{g}=\mathrm{Perm}(q_{g},r\prime^{\left(g\right)})$ (see the functions PrepQuery and ROT), the server computes *d* by following:

$$d={⊕}_{i=0}^{M}\mathrm{Enc}(r_{i}\times (q\prime_{f}[i]-q\prime_{g}[i]))$$

where *r_i_* is a random value. Dec(d) is equal to 0 iff. $q_{f}=q_{g}$. See Supplementary Algorithm S5 which defines a function isLongest. In addition to finding a set-longest match at *t*, it is convenient to find a longest substring which matches to at least *ε* sequences. This operation enables to avoid detecting unique haplotype and provides *ε*-anonymity result and is implemented by replacing the function: isLongest by another function: isLongestGT *ε* which computes flags each of which shows if the interval matches to 0,…,ε−1 respectively and returns shuffled flags, and the user knows the result by checking if there is a flag which is equal to zero. See Supplementary Algorithm S5 for more details.

The detailed algorithm of PBWT-sec is shown in Algorithm 2.

### 3.4 Concealing the search position

By the algorithm introduced above, the match position *t* needs to be provided to the server. Let us consider the case that *t* needs to be concealed (e.g. the used would not like to reveal which gene is analyzed). In practical genotype database search, it is often sufficient for the user to hide *t* in a set of multiple columns. Therefore, here we assume the following security model.

Model 3. *The user is a private haplotype sequence holder, and the server is a holder of a set of private haplotype sequences. The user has a vector of D positions* $T=(t_{1},\ldots ,t_{D})$*. The user learns nothing but a set-longest match at a given position* $t\in \{t_{1},\ldots ,t_{D}\}$ *between the query and the database while the server learns nothing about the user’s query string. The server knows T but cannot identify which element the user queries.*

Conceptually, the user could query multiple positions at the same time to conceal the search position. In the extreme case the user would query all search positions to avoid leaking any information about *t*. However, every answered query would leak more information from the database and querying would become computationally prohibitive. We therefore propose joint processing using OT that simultaneously uses multiple search positions. Let us define *V_c_* as another look-up vector for a letter *c* as follows:

$$\begin{array}{l} V_{c}[o_{j}+i]=\{\begin{array}{ll} CF_{c}(P_{\cdot ,(t_{j}+k)})+o_{j} & \left(i=0\right)\\ CF_{c}(P_{\cdot ,(t_{j}+k)})+\mathrm{Ran}k_{c}(P_{\cdot ,(t_{j}+k)},i)+o_{j} & \left(i\neq 0\right) \end{array}\\ \;(1\leq j\leq D,0\leq i\leq M) \end{array}$$

where $o_{j}=(j-1)(M+1)$ is an offset and *k* is an index which is initialized by –1 and incremented by 1 in each iteration of the recursive search. Note that ($V_{c}[o_{j}],\ldots ,V_{c}[o_{j}+M]$) corresponds to $v_{c}$ for *t_j_*th column. The algorithm for the Model 3 is designed by replacing the lookup tables $v_{c}$ by *V_c_* (see Step 2a, item 1 in Algorithm 2) and initializing *f* and *g* by *o_x_* and $o_{x}+M$, respectively, where *t *=* t_x_* (see Step 1 in Algorithm 2). As a result the tables get *D* times larger which has an impact on computing requirements and data transfer size (see Section 3.7). We therefore suggest using this algorithm for small *D*.

### 3.5 Reducing communication size

As we will describe in the Complexity analysis in the following section, the PBWT-sec algorithm using standard OT requires O(M|Σ|) in communication size per iteration in the best case, which makes the core algorithm less practical. We propose to use an algorithm for sublinear-communication OT (SC-OT) proposed in Zhang *et al.* (2013). Using this approach we can reduce the communication size of PBWT-sec to $O(\sqrt{M|\Sigma |})$ (best case). Here, we only outline the key ideas of SC-OT and its adaptation of PBWT-sec. In the SC-OT, the one encodes the position *t* by a two dimensional representation: $t_{0}=t\;/\left\lceil \sqrt{N}\;\right\rceil ,\;t_{1}={\left(t\right)}_{\text{mod}\lceil \sqrt{N}\rceil }$, where ⌈·⌉ denotes the ceil of the argument. The user sends $\mathrm{Enc}(t_{0})$ and $\vec{\mathrm{Enc}}(q)$ to the server, where

$$\vec{\mathrm{Enc}}(q)=(\mathrm{Enc}(q_{0}=0)\ldots ,\mathrm{Enc}(q_{t_{1}}=1),\ldots ,\mathrm{Enc}(q_{\left\lceil \sqrt{N}\right\rceil -1}=0)).$$

The server obtains random values$r_{k},k=0,\ldots ,\left\lceil \sqrt{N}\;\right\rceil -1$, and computes

$$c_{k}=\overset{\left\lceil \sqrt{N}\;\right\rceil -1}{\underset{i=0}{⊕}}(v[k\times \left\lceil \sqrt{N}\right\rceil +i]⊗\mathrm{Enc}(q_{i}))⊕(r_{k}⊗\mathrm{Enc}(t_{0}-k)),$$

and sends $c=(c_{0},\ldots ,c_{\left\lceil \sqrt{N}\;\right\rceil -1})$ to the user. The user knows the result by the decryption: $\mathrm{Dec}(c_{t_{0}})$. Note that $\mathrm{Enc}(t_{0}-k)=\mathrm{Enc}(0)$ iff. $t_{0}=k$, therefore the decryption of *c_i_* becomes a random value when $i\neq t_{0}$.

In order to apply bit-rotation technique naturally to SC-OT, the server needs to return v[t] in the same two dimensional representation. The key idea here is that the server creates $v_{0}$ and $v_{1}$ where $v_{0}[i]=v[i]/\left\lceil \sqrt{N}\;\right\rceil$ and $v_{1}[i]={\left(v[i]\right)}_{\text{mod}\lceil \sqrt{N}\;\rceil },\;i=0,\ldots ,N-1$, and searches on both $v_{0}$ and $v_{1}$. Similar to the linear communication size function ROT, the removable random factors are added to server’s returns. More details on SC-OT is given in Section S1. The complete algorithm for privacy-preserving search based on SC-OT is given in Supplementary and Algorithm S2.

### 3.6 An exhaustive baseline algorithm

There are a few related works in regard to finding a DNA substring match (Baldi *et al.*, 2011; Cristofaro *et al.*, 2013), however, the goal of PBWT-sec is to find the set-longest prefix match from a set of aligned sequences while those works aim to find a fixed-length approximate substring match between two sequences. Therefore, we will compare our algorithm with a baseline algorithm which can find the set-longest prefix match on the basis of exhaustive enumeration of *k*-mers. This baseline algorithm serves as a proxy for the other conceptually similar algorithms.

In order to identify the match, the user queries the server about the presence of a *k*-mer. Here, the server stores all *k*-mers, there are $O(|\Sigma {|}^{k})$ of them, and we use SC-OT. Such a strategy is efficient for short queries as $|\Sigma {|}^{k}$ is not too large. However, the resource requirements will be dominated by queries for large *k* and the algorithm quickly gets intractable.

### 3.7 Complexity

Most of the computing and transfer on server and user side is related to the encryption/decryption and the computational cost of the search is negligible. While PBWT requires essentially O(1) to update the intervals per iteration, PBWT-sec needs to conceal the query and requires M|Σ| operations on the server, where *M* is the number of sequences in the database and |Σ| is the size of the alphabet. When multiple queries are performed at the same time, i.e. *D* > 1, the effort increases linearly in *D*, i.e. the server sides compute effort is O(MD|Σ|) per iteration. When using SC-OT, the communication size and effort for the user is $O(\sqrt{MD|\Sigma |})$ (see Section 3.5 and Supplementary Section S1 for details).

Table 1 summarizes the time, data transfer overhead and space complexities of the PBWT-sec, when the server’s PBWT is *M *×* N* matrix consisting of a set of alphabet letters Σ and the user’s query length is ℓ and the number of queries positions is *D* (including *D* – 1 decoy positions; see Section 3.4 for details). For the purpose of comparison, we consider the method outlined in Section 3.6 that achieves the same security and utility as PBWT-sec. Since the complexity of the exhaustive approach is exponential to the query length, its performance deteriorates quickly for long matches. On the other hand, the time and data transfer overhead complexity of the PBWT-sec are linear and sub-linear to the query length, which enables the user to find a long match efficiently.

**Table 1.** The summary of the time, communication and space complexities of PBWT-sec (CP) and an exhaustive method (EX)is the length of query and is the alphabet size

	Time	Communication	Space
CP (user)	$O(\ell \sqrt{MD|\Sigma |})$	$O(\ell \sqrt{MD|\Sigma |})$	$O(\sqrt{MD|\Sigma |})$
CP (server)	O(ℓMD|Σ|)	$O(\ell \sqrt{MD|\Sigma |})$	O(MD|Σ|)
EX (user)	$O(\sqrt{D|\Sigma {|}^{\ell }})$	$O(\sqrt{D|\Sigma {|}^{\ell }})$	$O(\sqrt{D|\Sigma {|}^{\ell }})$
EX (server)	$O(D|\Sigma {|}^{\ell })$	$O(\sqrt{D|\Sigma {|}^{\ell }})$	$O(D|\Sigma {|}^{\ell })$

Both algorithms use SC-OT. *M* is the number of haplotype sequences (server side), *D* is the number of queried positions (including *D* – 1 decoy position to conceal the query position), ℓ is the length of query and |Σ| is the alphabet size.

### 3.8 Security notion

In this paper, we assume the security model called *Semi-honest model* where both parties follow the protocol, but an adversarial one attempts to infer additional information about the other party’s secret input from the legally obtained information. The semantic security of the encryption scheme used in the protocol (see Section 3.1) implies immediately that the server cannot infer any information about the user’s query q during the protocol. Also, the user cannot infer any information about server’s return except for the result.

Another security model is called *Malicious model* where an adversarial party cheats even in the protocol (e.g. by inputting maliciously chosen invalid values) in order to illegally obtain additional information about the secret. Here we briefly describe one example of an illegal access based on the Malicious model. In our protocol, the user needs to create a bit vector q of *N* that includes a bit that is 1 and the rest of the *N* – 1 bits are 0. If the malicious user creates a non-bit vector:

$$q_{k}=\{\begin{array}{ll} 1 & \left(k=i\right)\\ x & \left(k=j\right)\\ 0 & \left(k\neq i\wedge k\neq j\right) \end{array}\;$$

where *x* is a large integer, the server returns c=Enc(v[i]+x·v[j]). When *x* is larger than any element of v, the user can infer v[i] by (Dec(c))mod x and v[j] by Dec(c)/x. (For example, if *x* = 100 and Dec(c)=821, the user can detect v[i]=21 and v[j]=8.) Thus the server leaks two elements of v by a single query.

In this study, we do not discuss such cases in detail; however, we would like to mention that it is possible to design an algorithm for the Malicious model with a small modification. In order to avoid such attacks, the server needs to verify if the user sends a bit vector which includes only one bit that is 1 and rest of the bits are 0. To achieve this, we suggest using a cryptographic technique called Non-Interactive Zero Knowledge Proofs which enables the user to convince a server that each ciphertext Enc(m) corresponds to a value m ∈{0,1}, but does not leak any information about which of 0 and 1 is *m*. Among several algorithms, Sakai’s algorithm (Sakai *et al.*, 2013) has such a property. By using the algorithm, the server knows whether or not q[i]∈{0,1}. To return a correct result only if q includes only a single 1, it is sufficient for the server to add $w=r⊗(\mathrm{Enc}(q_{0})⊕\ldots ⊕\mathrm{Enc}(q_{N-1})⊕\mathrm{Enc}(-1))$ to the original result, where *r* is a random value. Note that w=Enc(0) iff. *q_i_* = 1 and *q_j_* = 0 for 0≤j<N and i≠j.

## 4 Experiments

In this section, we evaluate the performance of the proposed method on the datasets created from the chromosome 1 data from the 1000 Genomes Project phase 1 data release which consists of 2184 haploid genomes (The 1000 Genomes Project Consortium, 2012). In our experiments and as in Durbin (2014), we used alleles having SNPs, but we did consider indel variants. We used all 2184 genomes of original data for all the experiments.

We implemented the proposed algorithm in C ++ based on an open source C ++ library of elliptic curve ElGamal encryption provided by AIST. Our implementation supports communication over the network. We used the standard parameters called secp192k1 (SECG curve over a 192-bit prime field), according to the recommendation by The Standards for Efficient Cryptography Group. For comparison, we also implemented an exhaustive baseline method (see Section 3.6) that achieves the same security and utility as PBWT-sec. In order to perform a fair comparison, both PBWT-sec and the exhaustive method used the same SC-OT module where computation of *c_k_* (see Algorithm 1) is simply parallelized by OpenMP.

In the first experiment, the user selected a true start position together with 49 decoys (see Section 3.4 for details), and both PBWT-sec and the exhaustive method were run with the same computational setting: the user used a single thread of a laptop computer equipped with an Intel Core(TM) i7 3.00 GHz CPU and 16 GB memory, and the server used more than eight threads of another laptop equipped with an Intel Core(TM) i7 2.60 GHz CPU (four cores with hyper-threading) and 8 GB memory. Those two computers communicated over the network.

**Fig. 6.**
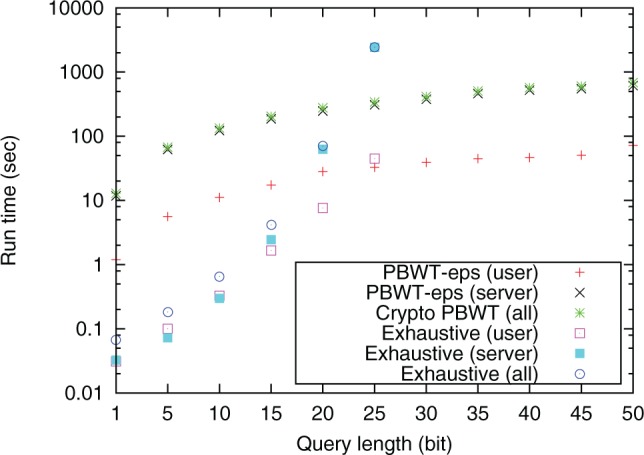
Run time of PBWT-sec and the exhaustive method on 2184 aligned haploid genomes on laptop computers equipped with four cores. The user selected 49 decoy positions for concealing the true query position. The server used all of the four cores with hyper-threading while the user used a single thread. ‘PBWT-sec (all)’ and ‘Exhaustive (all)’ include communication overhead

**Fig. 7.**
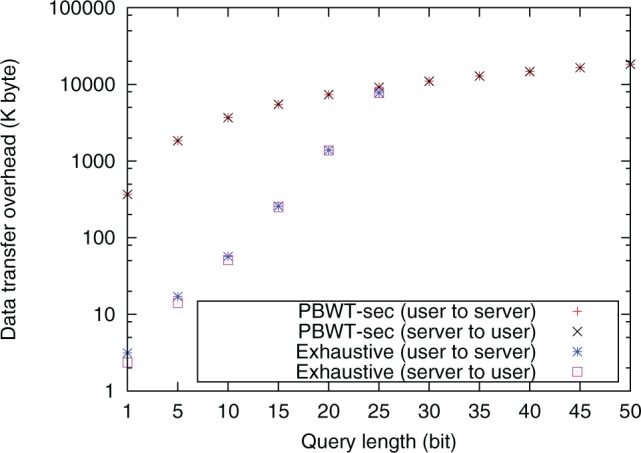
Data transfer overhead of PBWT-sec and the exhaustive method on 2184 aligned haploid genomes on laptop computers. The user selected 49 decoy positions for concealing the true query position

Figures 6 and 7 show run time and data transfer overhead of PBWT-sec and of the exhaustive method. The observed run time and data transfer size of PBWT-sec is linear in the query length, while that of the exhaustive approach is exponential. For query lengths larger than 30 bit, the computation of the exhaustive method did not finish within 24 h. These results fit the theoretical complexity described in Section 3.7. We also evaluated performance of the runtime of PBWT-sec when the user selected 0, 4, 9, 14, and 49 additional decoy positions. The search with a typical query of length 25 SNP positions and no decoy required no more than 15.5 s including communication overhead (Table 2)

**Table 2.** The run time of a typical query of length 25 SNP positions with PBWT-sec on *M *=* *2184 aligned haploid genomes on laptop computers equipped with four cores

	User	Server	All
Run time (sec) with *D* = 1	4.55	10.8	15.5
Run time (sec) with *D* = 5	8.77	34.0	43.0
Run time (sec) with *D* = 10	12.5	65.3	78.0
Run time (sec) with *D* = 20	17.3	124	142
Run time (sec) with *D* = 50	27.5	311	339

The server used all the four cores with hyper-threading while the user used a single thread. All included communication overhead. *D* is the number of positions queried simultaneously to conceal the query position (if required).

.

The user’s run time of PBWT-sec is relatively small, making it suitable for a practical case where computation power in a server side is generally stronger than that of user side. Since the memory usage of PBWT-sec does not depend on query length, it uses less than 60 MB while that of the exhaustive method exponentially increases according to the query length and required 6 GB when the query length is 25 bit.

Although the exhaustive method is efficient for short queries, we consider that PBWT-sec is more practical when taking into account that the bit length of a unique substring for a human genome is greater than 31 bits. Moreover, since there are large linkage blocks, even queries with more than 100 bits would not always lead to unique matches in the 1000 genomes data. Hence, the exhaustive search strategy would either not always be able to return a unique match or would be very inefficient. The proposed iterative privacy-preserving technique is efficient also for long queries.

In the second experiment, we evaluated the performance of the run time of PBWT-sec on a compute node equipped with four CPU sockets (Intel Xeon 2.40 GHz CPU; total of 32 cores with hyper-threading). In this experiment, the user also selected 0, 4, 9, 14 and 49 additional decoy positions. For environmental reasons, we did not perform communication over the network and the data was transferred by file I/O which is also included in run time.

Although the current implementation is a prototype and there is room for improvement in terms of parallelization, the server’s run time was at an acceptable level in practical configurations (Table 3). We note, that with improvements in parallelization, the server run time may be reduced to 3–4 s.

**Table 3.** The run time of a typical query with PBWT-sec on *M *=* *2184 aligned haploid genomes on a compute node with up to 16 cores with hyper-threading and a query length of 25 SNP positions

Parallel Compute Cores	4	8	16
Run time (sec) with *D* = 1	22.6	15.5	7.9
Run time (sec) with *D* = 5	47.3	40.0	18.4
Run time (sec) with *D* = 10	84.5	68.4	31.6
Run time (sec) with *D* = 20	154	114	56.5
Run time (sec) with *D* = 50	386	260	132.6

Wall time includes server (≈90%) and user time (≈10%). *D* is the number of positions queried simultaneously to conceal the query position (if required).

## 5 Conclusion

In this paper, we have proposed a novel approach for searching genomic sequences in a privacy-preserving manner. Our approach combines an efficient data structure that can be used for recursive search and a novel approach for recursive oblivious transfer. It achieves high utility and has strong security features and requires acceptable compute and communication resources.

The developed novel algorithm can find the longest match between a query and a large set of aligned genomic sequences indexed by PBWT. We implemented our algorithm and tested on the dataset created from the 1000 Genomes Project data (The 1000 Genomes Project Consortium, 2012). Compared to an exhaustive baseline approach, our algorithm, named PBWT-sec, was orders of magnitude more efficient both in run time and data transfer overhead for practical query sizes. When the prototype program was run on laptop machines, the total run time including communication time over the network was 15.5 s for searching on 2184 genomes without concealing the query position. Searches with a concealed query position using a compute node took between 18.6 and 133 s depending on the level of privacy.

As the original data structure supports many useful search options such as wild card search and set maximal search, PBWT-sec could also support those options by using the same techniques used in the original structures in combination with cryptographic techniques, including OT. Moreover, the approach could be easily applied for BWT and has a potential to be applied for other recursively searchable data structures.

To the best of our knowledge, the proposed algorithm is the first that allows set-maximal search of genomic sequences in a privacy-preserving manner for user and database. We note that the implementation can still be improved and the overall run time can likely be reduced to not more than a few seconds per query. This would make it practical to use our approach in a genomic Beacon (see GA4GH’s Beacon Project) that would allow the privacy-preserving search for combinations of variants. It also appears practical to use our approach to enable search by a user that has access to his/her genomic sequence and would like to query the database, for instance, for information related to disease risk without sharing this information with anybody. Finally, the algorithm can also be used to facilitate sharing of genetic information across institutions and countries in order to identify large enough cohorts with a similar genetic backgrounds. This is in spirit of the mission of the Global Alliance for Genome and Health.

## Supplementary Material

Supplementary Data
