# Supplementary material for: Efficient privacy-preserving string search and an application in genomics
Source: Bioinformatics. 2016 Mar 2;32(11):1652–61. doi: 10.1093/bioinformatics/btw050 (PMC4892414; doi:10.1093/bioinformatics/btw050)
Supplement: Supplementary Data [file supp_32_11_1652__index.html]

Efficient Privacy-Preserving String Search and an Application in Genomics — Efficient privacy-preserving string search and an application in genomics — Efficient privacy-preserving string search and an application in genomics — Supplementary Data 

# Efficient privacy-preserving string search and an application in genomics

## Supplementary Data

files

- Supplementary Data - pdf file
