# Supplementary material for: Efficient privacy-preserving string search and an application in genomics
Source: Bioinformatics. 2016 Mar 2;32(11):1652–61. doi: 10.1093/bioinformatics/btw050 (PMC4892414; doi:10.1093/bioinformatics/btw050)
Supplement: Supplementary Data [file supp_btw050_bioinfo_supplement_clean.pdf]

Supplementary Material for "An Efficient Privacy-Preserving Algorithm for String Search and its Application to Genomics"

Kana Shimizu[1,4,*] Koji Nuida[2,3] Gunnar Rätsch[4,*]

[1] Biotechnology Research Institute for Drug Discovery
National Institute of Advanced Industrial Science and Technology,
2-4-7 Aomi Koto-ku, Tokyo 135-0064, Japan,
[2] Information Technology Research Institute,
National Institute of Advanced Industrial Science and Technology,
2-4-7 Aomi Koto-ku, Tokyo 135-0064, Japan,
[3] Japan Science and Technology Agency (JST) PRESTO Researcher,
Tokyo, Japan
[4] Computational Biology, Memorial Sloan Kettering Cancer Center,
1275 York, New York, NY, USA

S1 The sublinear communication size recursive oblivious transfer

In this section, we describe the detailed algorithm of the sublinear communication size recursive oblivious transfer. In Section 3.2, we introduced the bit-rotation technique for the case of the linear communication size oblivious transfer. As mentioned in Section 3.5, the same technique is also applied for the $O(\sqrt{N})$-communication size oblivious transfer (SC-OT).

S1.1 The sublinear communication size oblivious transfer

Let us review the SC-OT algorithm. In the SC-OT, the one encodes the position t by in a two dimensional representation: $t_0 = t\ / \lceil \sqrt{N}\ \rceil$, $t_1 = (t)_{\mathsf{mod} \lceil \sqrt{N}\ \rceil}$, where $\lceil \cdot \rceil$ denotes the ceil of the argument. The user sends $\mathsf{Enc}(t_0)$ and $\vec{\mathsf{Enc}}(\boldsymbol{q})$ to the server, where

$$\vec{\mathsf{Enc}}(\boldsymbol{q}) = (\mathsf{Enc}(q_0 = 0) \ldots, \mathsf{Enc}(q_{t_1} = 1), \ldots, \mathsf{Enc}(q_{\lceil \sqrt{N}\ \rceil - 1} = 0)).$$

The server obtains random values r_k for $k = 0, \ldots, \lceil \sqrt{N}\ \rceil - 1$, and computes

$$c_k = \bigoplus_{i=0}^{\lceil \sqrt{N}\ \rceil - 1} \big(v[k \times \lceil \sqrt{N}\ \rceil + i] \otimes \mathsf{Enc}(q_i)\big) \oplus \big(r_k \otimes \mathsf{Enc}(t_0 - k)\big),$$

and sends $\boldsymbol{c} = (c_0, \ldots, c_{\lceil \sqrt{N}\ \rceil - 1})$ to the user. The user knows the result by the decryption: $\mathsf{Dec}(c_{t_0})$. Note that $\mathsf{Enc}(t_0 - k) = \mathsf{Enc}(0)$ iff. $t_0 = k$, therefore the decryption of c_i becomes a random value when $i \neq t_0$. See the function SCOT in Algorithm S1 for detailed description.

S1.2 Bit-rotation technique for the sublinear communication size oblivious transfer

In this section, we will describe a new algorithm for the sublinear communication size recursive oblivious transfer (SC-ROT) by using the bit-rotation technique which is introduced in the main text. In order to apply bit-rotation technique naturally to SC-OT, the server needs to return $v[t]$ in the same two dimensional representation. The key idea here is that the server creates $\boldsymbol{v}_0$ and $\boldsymbol{v}_1$ where $v_0[i] = v[i] / \lceil \sqrt{N}\ \rceil$ and $v_1[i] = (v[i])_{\mathsf{mod} \lceil \sqrt{N}\ \rceil}$, $i = 0, \ldots, N-1$, and searches on both $\boldsymbol{v}_0$ and $\boldsymbol{v}_1$. The user obtains next t_0 and t_1 in randomized form by the search on $\boldsymbol{v}_0$ and $\boldsymbol{v}_1$ respectively using the same $\mathsf{Enc}(t_0)$ and $\vec{\mathsf{Enc}}(\boldsymbol{q})$. For the search on $v_0[i]$, the server generates random value $r_0 \in \{0, \ldots, \lceil \sqrt{N}\ \rceil - 1\}$ and returns an encrypted value of $\hat{t_0} = (v_0[i] + r_0)_{\mathsf{mod} \lceil \sqrt{N}\ \rceil}$. For the search on $v_1[i]$, the server generates random value $r_1 \in \{0, \ldots, \lceil \sqrt{N}\ \rceil - 1\}$ and returns an encrypted value of $\hat{t_1} = (v_1[i] + r_1)_{\mathsf{mod} \lceil \sqrt{N}\ \rceil}$. The user decrypts the server's return and obtains $\hat{t_0}$ and $\hat{t_1}$ to generate the next query $\mathsf{Enc}(\hat{t_0})$ and $\vec{\mathsf{Enc}}(\hat{\boldsymbol{q}}) = (\mathsf{Enc}(\hat{q}_0 = 0) \ldots, \mathsf{Enc}(\hat{q}_{\hat{t_1}} = 1), \ldots, \mathsf{Enc}(\hat{q}_{\lceil \sqrt{N}\ \rceil - 1} = 0))$. Since the server

*to whom correspondence should be addressed

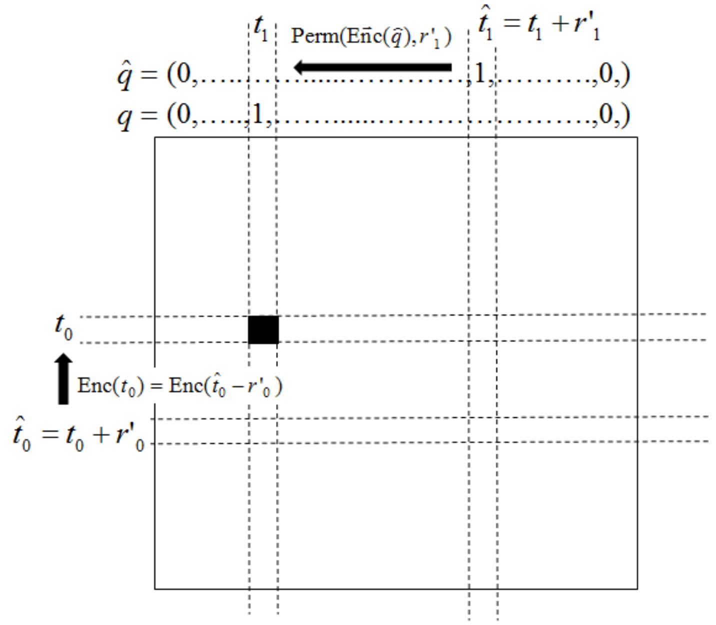

Figure S1: The illustration for the removal of random factors in the server side. $\boldsymbol{q}$ and t_0 show the plain text of the user's 'true' query while $\hat{\boldsymbol{q}}$ and $\hat{t_0}$ show the plain text of the user's query. The server recovers correct t_1 by computing -r'_1 rotated permutation of the server's query $\hat{\boldsymbol{q}}$. It also recovers correct t_0 by the homomorphic encryption: $\mathsf{Enc}(\hat{t_0} - r'_0)$.

knows r_0 and r_1, he/she can remove those random factors by $\mathsf{Enc}(\hat{t_0} - r_0)$ and the circular bit permutation $\mathsf{Perm}(\hat{\boldsymbol{q}},\ r_1)$ before conducting the next search on $\boldsymbol{v}_0$ and $\boldsymbol{v}_1$. To implement such property for the server side, we designed the server's function SCROT which is described in Algorithm S1. It takes nine arguments: user's query $\mathsf{Enc}(\hat{t_0})$, $\vec{\mathsf{Enc}}(\hat{\boldsymbol{q}})$, a vector to be searched $\boldsymbol{v}_x$ ($x \in \{0,1\}$), a random value r for randomizing the result, upper bound of the true value L_x ($x \in \{0,1\}$), random values r'_0 and r'_1 which were used for randomizing 'true' values t_0 and t_1 in the previous round (i.e., $\hat{t_0} = t_0 + r'_0$ and $\hat{t_1} = (t_1 + r'_1)_{\mathsf{mod} L_1}$) and row length L_0 and column length L_1 of the two dimensional representation (i.e., $L_0 = L_1 = \lceil\sqrt{N}\,\rceil$ for this case). Figure S1 illustrates the server process for removing random factors previously added to the server's return. Since $\mathsf{Enc}(\hat{t_0} - r'_0)$ causes the position shift from $\hat{t_0}$ to $(\hat{t_0} - r'_0)_{\mathsf{mod} L_0}$ in server's return $\boldsymbol{c}$, the server also needs another permutation $\mathsf{Perm}(\boldsymbol{c},\ -r'_0)$ before returning the result. See Algorithm S1 for detailed description. By this function SCROT, the server can add *removable* random factor to the result, and therefore it enables user to search $\boldsymbol{v}$ recursively.

S1.3 Solving the problem caused by modulo operation of $v[i] + r$

In the function SCROT, the server generates random value $r \in \{0, \ldots, \lceil\sqrt{N}\,\rceil - 1\}$ and conducts randomization by:

$$(v[i] + r)_{\mathsf{mod}\lceil\sqrt{N}\,\rceil},$$

and returns $\mathsf{Enc}((v[i] + r)_{\mathsf{mod}\lceil\sqrt{N}\,\rceil})$ to the user.

Since the modulo operation yields different results for the same r according to the two conditions:

$$v[i] + r < \lceil\sqrt{N}\,\rceil$$

and

$$v[i] + r \geq \lceil\sqrt{N}\,\rceil\ ,$$

and neither the user nor the server knows which condition is applied (note that the user's choice $v[i]$ and server's random value are their private information), the server needs to return two results assuming both conditions in the next round. For the case of computing $\mathsf{Enc}(t_0)$, the sever needs to compute both

$$\boldsymbol{c}_0 \leftarrow \mathsf{SCROT}(\mathsf{Enc}(t_0), \vec{\mathsf{Enc}}(\boldsymbol{q}), \boldsymbol{v}_0, r_0, \lceil\sqrt{N}\,\rceil, r'_0, r'_1, \lceil\sqrt{N}\,\rceil, \lceil\sqrt{N}\,\rceil)$$

and

$$\boldsymbol{c}'_0 \leftarrow \mathsf{SCROT}(\mathsf{Enc}(t_0), \vec{\mathsf{Enc}}(\boldsymbol{q}), \boldsymbol{v}_0, r_0, \lceil\sqrt{N}\,\rceil, (r'_0 - \lceil\sqrt{N}\,\rceil), r'_1, \lceil\sqrt{N}\,\rceil, \lceil\sqrt{N}\,\rceil)\ .$$

Since only one of c_{0,t_0} and c'_{0,t_0} becomes an encryption of a correct result and the other becomes an encryption of a random value, user is able to obtain the next t_0 by checking if $0 \leq \mathsf{Dec}(c_{0,t_0}) < \lceil\sqrt{N}\,\rceil$ or $0 \leq \mathsf{Dec}(c'_{0,t_0}) < \lceil\sqrt{N}\,\rceil$ (see the function: $\mathsf{SCChooseDec}$ in Algorithm S1). In similar way, the user also obtains t_1. Algorithm S2 shows the full description of sublinear communication size recursive oblivious transfer algorithm taking into account of the above problem.

S2 The sublinear communication algorithm for $PBWT - sec$

In Section 3.3, the linear size communication algorithm for $PBWT - sec$ is introduced. Here we introduce the sublinear communication size algorithm by adapting SC-ROT to the search by $PBWT$. The goal is to find a set-longest match at a given position t between a query S and a set of genotype sequences X in a privacy-preserving manner. in this section, we consider that both t and S are private information and use the following model which is the same model as Model 3 in Section 3.4.

Model[n1] 1. *The user is a private haplotype sequence holder, and the server is a holder of a set of private haplotype sequences. The user has a vector of D positions $T = (t_1, \ldots, t_D)$. The user learns nothing but a set-longest match at a given position $t \in \{t_1, \ldots, t_D\}$ between the query and the database while the server learns nothing about the user's query string. The server knows T but cannot identify which element the user queries.*

Similar to the linear size communication algorithm for $PBWT - sec$, the server creates $\boldsymbol{v}^{(c)}$ which is a look-up vector for a letter c as follows:

$$v^{(c)}[o_j + i] = \begin{cases} \mathsf{CF}_c(P_{\cdot,(t_j+k)}) + o_j & (i = 0) \\ \mathsf{CF}_c(P_{\cdot,(t_j+k)}) + \mathsf{Rank}_c(P_{\cdot,(t_j+k)}, i) + o_j & (i \neq 0) \end{cases}$$
$$(1 \leq j \leq D, 0 \leq i \leq M)$$

where $o_j = (j-1)(M+1)$ is an offset and k is an index which is initialized by -1 and incremented by 1 in each iteration of recursive search. All those letter tables $\boldsymbol{v}^c$ for $c \in \Sigma$ are concatenated into one single vector $\boldsymbol{v}$ to minimize data transfer overhead. When updating the interval to extend matches by a letter $S[i]$, the user needs to specify the region of $\boldsymbol{v}$, which corresponds to a letter table $\boldsymbol{v}^{(S[i])}$. In our algorithm, we designed row length L_0 and column length L_1 for the two dimensional representation (L_0 and L_1 are not the matrix size of PBWT) such that elements of the same position in the different letter tables should be placed in the same column after concatenating all the tables (i.e., $(i)_{\mathsf{mod} L_1}$ $= (i + |\boldsymbol{v}^{(0)}|)_{\mathsf{mod} L_1} = (i + |\boldsymbol{v}^{(0)}| + |\boldsymbol{v}^{(1)}|)_{\mathsf{mod} L_1}, \ldots, = (i + \sum_{c \in \{0,\ldots,|\Sigma|-2\}} |\boldsymbol{v}^{(c)}|)_{\mathsf{mod} L_1}$) in order that the user can specify the letter table by choosing an offset added to row value (i.e., $\hat{t_0}$) of the query. For this purpose, the server configures $L_1 = \sqrt{D(M+1)|\Sigma|}$, an offset factor $L_0' = \lceil D(M+1)/L_1 \rceil$, $L_0 = L_0' \times |\Sigma|$, and extend each letter table $\boldsymbol{v}^{(c)}$ to the length of $L_0' L_1$ before the concatenation to make $\boldsymbol{v}$ (i.e., $|\boldsymbol{v}^{(0)}| =, \ldots, = |\boldsymbol{v}^{(|\Sigma|-1)}| = L_0' L_1$). To enable searching $\boldsymbol{v}^{(c)}$ by SC-ROT, the server converts all the elements in $\boldsymbol{v}$ into the two dimensional representation and stores them in two vectors $\boldsymbol{v}_0$ and $\boldsymbol{v}_1$ each of them is of length $L_0' L_1$. Figure S2 is a graphical view of the rearrangement of $\boldsymbol{v}_0$ and $\boldsymbol{v}_1$.

Now the user is able to search $\boldsymbol{v}^{(c)}$ recursively in an oblivious manner by using SC-ROT. In PBWT, the match is reported as an interval $(f, g]$ and the number of matches is equivalent to $g - f$. Since the user wants to start the search from t_x-th column on PBWT, user initialized f and g by $f = o_x$, $g = o_x + M$ where $o_j = (j-1)(M+1)$ and computes two dimensional representation of them: $f_0 = f/L_1$, $f_1 = (f)_{\mathsf{mod} L_1}$, $g_0 = g/L_1$, $g_1 = (g)_{\mathsf{mod} L_1}$. Then the user recursively searches $\boldsymbol{v}^{(c)}$ for updating f and g until he/she finds the match. For the i-th round of the recursive search, meaning that the user updates the interval for finding matches ending with $S[i]$, he/she adds an offset $S[i]L_0'$ to f_0 and g_0 in order to specify $S[i]$. For each round, the server also computes an encrypted flag whose plain text is equal to 0 iff. $f = g$. Since there is also a similar problem caused by modulo operation discussed in the section S1.3, the server computes the encrypted flag for the case of $v[t] + r < L_0$ and $v[t] + r \geq L_0$. The detailed description of this part is described in the function $\mathsf{isSCLongest}$ in Algorithm S3 and item 3-(b) of Algorithm S4. Finally, the user learns the set-longest match at t by $\mathsf{Dec}(d)$. In order to hide the length of the set-longest match to the server, the user keep sending decoy queries until it reaches to ℓ-th round. Algorithm S3 and Algorithm S4 show a detailed algorithm of *PBWT-sec*.

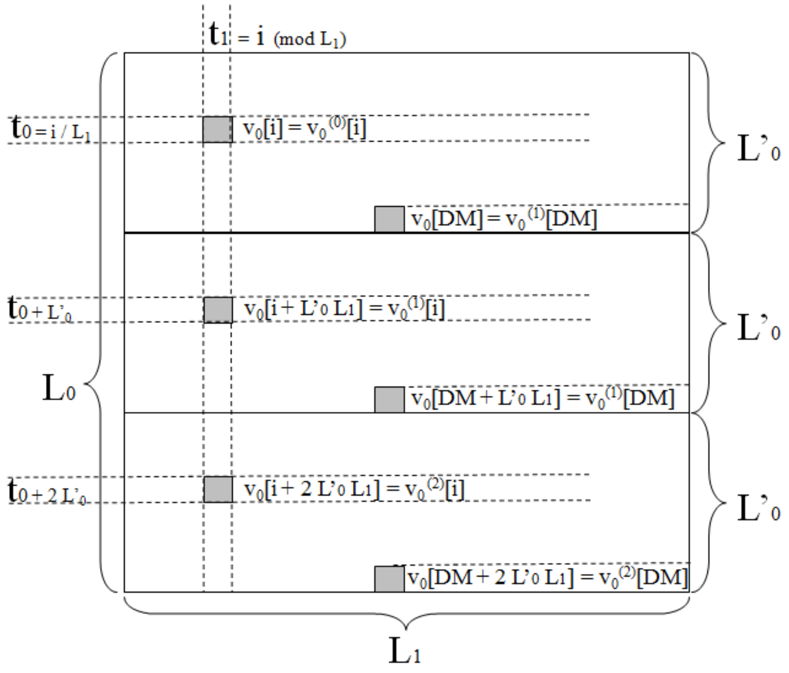

Figure S2: The arrangement of elements of $\boldsymbol{v}_0$ when $\Sigma = \{0, 1, 2\}$. The length of $\boldsymbol{v}_0^{(c)}$ for $c \in \Sigma$ is designed such that $v_0^{(0)}[i]$, $v_0^{(1)}[i]$ and $v_0^{(2)}[i]$ are aligned in the same column after the concatenation. The elements of $\boldsymbol{v}_1$ is also arranged in the same manner.

Algorithm S1 Building blocks for sublinear communication size recursive oblivious transfer and $PBWT - sec$

function SCPrepQuery(t_0, t_1, L_1)
    $\boldsymbol{q} = (q_0 = 0, \ldots, q_{t_1} = 1, \ldots, q_{L_1 - 1} = 0)$
    $\vec{\mathsf{Enc}}(\boldsymbol{q}) = (\mathsf{Enc}(q_1) \ldots, \mathsf{Enc}(q_{L_1}))$
    return $\mathsf{Enc}(t_0)$, $\vec{\mathsf{Enc}}(\boldsymbol{q})$
end function

function SCOT($\mathsf{Enc}(t_0)$, $\vec{\mathsf{Enc}}(\boldsymbol{q})$, $\boldsymbol{v}$, L_0, L_1)
    for $k = 0$ to $L_0 - 1$ **do**
        Generate random value r_k
        $x = k \times L_1$
        $c_k = \bigoplus_{i=0}^{L_1 - 1} \left(v[x + i] \otimes \mathsf{Enc}(q_i) \right) \oplus r_k \otimes \mathsf{Enc}(t_0 - k)$
    end for
    return $\boldsymbol{c} = (c_0, \ldots, c_{L_0 - 1})$
end function

function SCROT($\mathsf{Enc}(\hat{t_0})$, $\vec{\mathsf{Enc}}(\hat{\boldsymbol{q}})$, $\boldsymbol{v}$, r, L, r'_0, r'_1, L_0, L_1)
    $\hat{\boldsymbol{v}} \leftarrow (\boldsymbol{v} + r)_{\mathsf{mod} L}$
    $\boldsymbol{c} \leftarrow$ SCOT($\mathsf{Enc}(\hat{t_0} - r'_0)$, $\mathsf{Perm}(\vec{\mathsf{Enc}}(\hat{\boldsymbol{q}}),\ r'_1)$, $\hat{\boldsymbol{v}}$, L_0, L_1)
    $\boldsymbol{c} \leftarrow \mathsf{Perm}(\boldsymbol{c}, -r'_0)$ ▷ recovering the original position
    return $\boldsymbol{c} = (c_0, \ldots, c_{L_0 - 1})$
end function

function SCChooseDec(c_0, c_1, L)
    for $x = 0$ to 1 **do**
        $m \leftarrow \mathsf{Dec}(c_x)$
        if $(0 \leq m < L)$
            return m
        end if
    end for
end function

Algorithm S2 The detailed protocol of the sublinear communication size recursive oblivious transfer.

- Public input: the database size N, query length ℓ
- Private input of a user: a start position $t \in 0, \dots, N-1$
- Private input of a server: a vector $\boldsymbol{v}$ of length N

0. (*Key setup of cryptosystem*) The user generates a key pair $(\mathsf{pk}, \mathsf{sk})$ by the key generation algorithm KeyGen for the additive-homomorphic cryptosystem and sends public key pk to the server (the user and the server share public key pk and only the user knows secret key sk).
1. (*Server initialization*) The server computes $v_0[i] = v[i]/\lceil\sqrt{N}\,\rceil$, $v_1[i] = (v[i])_{\mathsf{mod}\lceil\sqrt{N}\,\rceil}$ for $i = 0, \dots, N-1$.
2. (*User initialization*) The user computes $t_0 = t/\lceil\sqrt{N}\,\rceil$, $t_1 = (t)_{\mathsf{mod}\lceil\sqrt{N}\,\rceil}$.
3. (*Recursive search*)
   Initializes the index by $i \leftarrow 1$

while $(i \le \ell)$ **do**

(a) (*Query entry*) The user performs the following steps:

- Prepare query
  if $(i \ne 1)$
  $t_0 \leftarrow \mathsf{Dec}(c_{0,t_0})$, $t_1 \leftarrow \mathsf{Dec}(c_{1,t_0})$

end if

$\mathsf{Enc}(t_0)$, $\vec{\mathsf{Enc}}(\boldsymbol{q}) \leftarrow \mathsf{SCPrepQuery}(t_0, t_1, \lceil\sqrt{N}\,\rceil)$

- Sending $\mathsf{Enc}(t_0)$, $\vec{\mathsf{Enc}}(\boldsymbol{q})$ to the server.

(b) (*Searching*) The server performs the following steps:

if $(i \ne \ell)$
  Generating random values $r_0, r_1 \in \{0, \dots, \lceil\sqrt{N}\,\rceil - 1\}$
else
  $r_0 = 0$, $r_1 = 0$
end if
  ▷ ROT removes r'_0, r'_1 from a query and add r_0 or r_1 to each result.
$\boldsymbol{c}_0 \leftarrow \mathsf{SCROT}(\mathsf{Enc}(t_0), \vec{\mathsf{Enc}}(\boldsymbol{q}), \boldsymbol{v}_0, r_0, \lceil\sqrt{N}\,\rceil, r'_0, r'_1, \lceil\sqrt{N}\,\rceil, \lceil\sqrt{N}\,\rceil)$
$\boldsymbol{c}'_0 \leftarrow \mathsf{SCROT}(\mathsf{Enc}(t_0), \vec{\mathsf{Enc}}(\boldsymbol{q}), \boldsymbol{v}_0, r_0, \lceil\sqrt{N}\,\rceil, (r'_0 - \lceil\sqrt{N}\,\rceil), r'_1, \lceil\sqrt{N}\,\rceil, \lceil\sqrt{N}\,\rceil)$
$\boldsymbol{c}_1 \leftarrow \mathsf{SCROT}(\mathsf{Enc}(t_0), \vec{\mathsf{Enc}}(\boldsymbol{q}), \boldsymbol{v}_1, r_1, \lceil\sqrt{N}\,\rceil, r'_0, r'_1, \lceil\sqrt{N}\,\rceil, \lceil\sqrt{N}\,\rceil)$
$\boldsymbol{c}'_1 \leftarrow \mathsf{SCROT}(\mathsf{Enc}(t_0), \vec{\mathsf{Enc}}(\boldsymbol{q}), \boldsymbol{v}_1, r_1, \lceil\sqrt{N}\,\rceil, (r'_0 - \lceil\sqrt{N}\,\rceil), r'_1, \lceil\sqrt{N}\,\rceil, \lceil\sqrt{N}\,\rceil)$
$r'_0 \leftarrow r_0$, $r'_1 \leftarrow r_1$

- Sending $\boldsymbol{c}_0$, $\boldsymbol{c}'_0$, $\boldsymbol{c}_1$, $\boldsymbol{c}'_1$ to the user.

$i \leftarrow i + 1$

end while

4. (*Decryption of the result*) The user performs the following steps to obtain result x.
   $t_0 \leftarrow \mathsf{SCChooseDec}(c_{0,t_0}, c'_{0,t_0}, \lceil\sqrt{N}\,\rceil)$, $t_1 \leftarrow \mathsf{SCChooseDec}(c_{1,t_0}, c'_{1,t_0}, \lceil\sqrt{N}\,\rceil)$
   $x = t_0 \times \lceil\sqrt{N}\,\rceil + t_1$

Algorithm S3 Building blocks for sublinear communication size $PBWT-sec$

function isSCLongest($\mathsf{Enc}(f_0)$, $\mathsf{Enc}(g_0)$, $\vec{\mathsf{Enc}}(\boldsymbol{q}_f)$, $\vec{\mathsf{Enc}}(\boldsymbol{q}_g)$)
  for $i = 0$ to $L_1 - 1$ **do**
    Generating random value r
    $d = d \oplus \mathsf{Enc}\big(r \times (q_f[i] - q_g[i])\big)$
  end for
  Generating random value r
  $d = d \oplus \mathsf{Enc}\big(r \times (f_0 - g_0)\big)$
  return d
end function

function isSCLongestGTϵ($\mathsf{Enc}(f_0)$, $\mathsf{Enc}(g_0)$, $\vec{\mathsf{Enc}}(\boldsymbol{q}_f)$, $\vec{\mathsf{Enc}}(\boldsymbol{q}_g)$, ϵ)
  for $k = 0$ to $\epsilon - 1$ **do** ▷ For the case that $q_g[i] = 1$ move to $q'_g[j]$ when $i > j$
    $\vec{\mathsf{Enc}}(\boldsymbol{q}'_g) = \mathsf{Perm}(\vec{\mathsf{Enc}}(\boldsymbol{q}_g), k)$ ▷ $\boldsymbol{q}'_f = \mathsf{Perm}(\boldsymbol{q}'_g, k)$ iff. $(g - f) = k$
    for $i = L_1 - k$ to $L_1 - 1$ **do**
      $\mathsf{Enc}(q'_g[i]) = \mathsf{Enc}(0)$ ▷ Avoid a wrong match
    end for
    for $i = 0$ to $L_1 - 1$ **do**
      Generating random value r
      $d = d \oplus \mathsf{Enc}\big(r \times (q'_f[i] - q'_g[i])\big)$
    end for
    Generating random value r
    $d_k = d \oplus \mathsf{Enc}\big(r \times (f_0 - g_0)\big)$
  end for

  for $k = 0$ to $\epsilon - 1$ **do** ▷ For the case that $q_g[i] = 1$ move to $q'_g[j]$ when $i < j$
    $\vec{\mathsf{Enc}}(\boldsymbol{q}'_g) = \mathsf{Perm}(\vec{\mathsf{Enc}}(\boldsymbol{q}_g), k)$ ▷ $\boldsymbol{q}'_f = \mathsf{Perm}(\boldsymbol{q}'_g, k)$ iff. $(g - f) = k$
    for $i = 0$ to k **do**
      $\mathsf{Enc}(q'_g[i]) = \mathsf{Enc}(0)$ ▷ Avoid a wrong match
    end for
    for $i = 0$ to $L_1 - 1$ **do**
      Generating random value r
      $d = d \oplus \mathsf{Enc}\big(r \times (q'_f[i] - q'_g[i])\big)$
    end for
    Generating random value r
    $d_{\epsilon+k} = d \oplus \mathsf{Enc}\big(r \times (f_0 - g_0 + 1)\big)$
  end for
  $\boldsymbol{d} = (d_0, \ldots, d_{2\epsilon})$
  Shuffling order of elements in $\boldsymbol{d}$
  return $\boldsymbol{d}$
end function

Algorithm S4 The detailed description of sublinear communication size $PBWT - sec$ for finding a set-longest match at position t.

- Public input: The length of column M, a set of alphabet letters $\Sigma = \{0, 1, .., |\Sigma| - 1\}$ and a set of $(D-1)$ decoy positions and true position $T = (t_1, \ldots, t_D)$.
- Private input of a user: A starting column $t_x \in T$, a query sequence S of length ℓ
- Private input of a server: PBWT matrix $P \in \mathbb{N}^{M \times N}$

0. (*Key setup of cryptosystem*) The user generates a key pair $(\mathsf{pk}, \mathsf{sk})$ by the key generation algorithm KeyGen for the additive-homomorphic cryptosystem and sends public key pk to the server (while only the user knows secret key sk).
1. (*Server initialization*)
   - The server computes $L_1 = \sqrt{D(M+1)|\Sigma|}$, $L'_0 = \lceil D(M+1)/L_1 \rceil$, $L_0 = L'_0 \times |\Sigma|$ and announces L_0, L_1 and L'_0 to the user.
2. (*User initialization*)
   - The user initialize a half-open interval $(f, g]$ by $f = o_x,\ g = o_x + M$ where $o_j = (j-1)(M+1)$.
   - The user computes two dimensional representation of $(f, g]$ by $f_0 \leftarrow f/L_1,\ f_1 \leftarrow (f)_{\mathsf{mod} L_1},\quad g_0 \leftarrow g/L_1,\ g_1 \leftarrow (g)_{\mathsf{mod} L_1}$
3. (*Recursive search*) Initializes the indices by $i \leftarrow 1 \quad k \leftarrow -1$

while $(i \leq \ell)$ **do**

(a) (*Query entry*) The user performs the following steps:

- Prepare next query:
  $f_0 \leftarrow f_0 + S[i]L'_0,\ g_0 \leftarrow g_0 + S[i]L'_0$ ▷ Setting offset to search matches ending with $S[i]$
  $\big(\mathsf{Enc}(f_0),\ \vec{\mathsf{Enc}}(\boldsymbol{q}_f)\big) \leftarrow \mathsf{SCPrepQuery}(f_0,\ f_1,\ L_1)$, $\big(\mathsf{Enc}(g_0),\ \vec{\mathsf{Enc}}(\boldsymbol{q}_g)\big) \leftarrow \mathsf{SCPrepQuery}(g_0,\ g_1,\ L_1)$
- Sending $\mathsf{Enc}(f_0)$, $\vec{\mathsf{Enc}}(\boldsymbol{q}_f)$, $\mathsf{Enc}(g_0)$, $\vec{\mathsf{Enc}}(\boldsymbol{q}_g)$, $\mathsf{Enc}(S[i])$ to the server.

(b) (*Searching*) The server performs the following steps:

- Computes vectors $\boldsymbol{v}^{(c)}$ of length $D \times (M+1)$ for all $c \in \Sigma$:

$$v^{(c)}[o_j + u] = \begin{cases} \mathsf{CF}_c(P_{\cdot,(t_j+k)}) + o_j & (u = 0) \\ \mathsf{CF}_c(P_{\cdot,(t_j+k)}) + \mathsf{Rank}_c(P_{\cdot,(t_j+k)}, u) + o_j & (1 \leq u \leq M) \end{cases}$$

  where $o_j = (j-1)(M+1)$ for $j = 1, \ldots, D$.
- Creates vectors $\boldsymbol{v}_0^{(c)}, \boldsymbol{v}_1^{(c)}$ of length $L'_0 \times L_1$ for $c = 0, \ldots, |\Sigma| - 1$.
- Computes $v_0^{(c)}[i] = v^{(c)}[i]/L_1,\ v_1^{(c)}[i] = (v^{(c)}[i])_{\mathsf{mod} L_1}$ for $i = 0, \ldots, D(M+1) - 1$ and $c = 0, \ldots, |\Sigma| - 1$.
- Creates vectors $\boldsymbol{v}_0$ and $\boldsymbol{v}_1$ by concatenating $\boldsymbol{v}_0 = \boldsymbol{v}_0^{(0)}, \ldots, \boldsymbol{v}_0^{(|\Sigma|-1)}$ and $\boldsymbol{v}_1 = \boldsymbol{v}_1^{(0)}, \ldots, \boldsymbol{v}_1^{(|\Sigma|-1)}$.
- Generates random values $r_0^{(f)}, r_0^{(g)} \in \{0, \ldots, L_0 - 1\}$, $r_1^{(f)}, r_1^{(g)} \in \{0, \ldots, L_1 - 1\}$
- Computes next intervals and an encrypted flag showing if the match is the longest

$$\begin{aligned}
\boldsymbol{c}_0^{(f)} &\leftarrow \mathsf{SCROT}^{\dagger}(\mathsf{Enc}(f_0),\ \vec{\mathsf{Enc}}(\boldsymbol{q}_f),\ \boldsymbol{v}_0,\ r_0^{(f)}, L_0,\ r_0'^{(f)},\ r_1'^{(f)},\ L_0,\ L_1),\\
\boldsymbol{c'}_0^{(f)} &\leftarrow \mathsf{SCROT}^{\dagger}(\mathsf{Enc}(f_0),\ \vec{\mathsf{Enc}}(\boldsymbol{q}_f),\ \boldsymbol{v}_0,\ r_0^{(f)}, L_0,\ (r_0'^{(f)} - L_0),\ r_1'^{(f)},\ L_0,\ L_1),\\
\boldsymbol{c}_1^{(f)} &\leftarrow \mathsf{SCROT}^{\dagger}(\mathsf{Enc}(f_0),\ \vec{\mathsf{Enc}}(\boldsymbol{q}_f),\ \boldsymbol{v}_1,\ r_1^{(f)}, L_1,\ r_0'^{(f)},\ r_1'^{(f)},\ L_0,\ L_1),\\
\boldsymbol{c'}_1^{(f)} &\leftarrow \mathsf{SCROT}^{\dagger}(\mathsf{Enc}(f_0),\ \vec{\mathsf{Enc}}(\boldsymbol{q}_f),\ \boldsymbol{v}_1,\ r_1^{(f)}, L_1,\ (r_0'^{(f)} - L_0),\ r_1'^{(f)},\ L_0,\ L_1),\\
\boldsymbol{c}_0^{(g)} &\leftarrow \mathsf{SCROT}^{\dagger}(\mathsf{Enc}(g_0),\ \vec{\mathsf{Enc}}(\boldsymbol{q}_g),\ \boldsymbol{v}_0,\ r_0^{(g)}, L_0,\ r_0'^{(g)},\ r_1'^{(g)},\ L_0,\ L_1),\\
\boldsymbol{c'}_0^{(g)} &\leftarrow \mathsf{SCROT}^{\dagger}(\mathsf{Enc}(g_0),\ \vec{\mathsf{Enc}}(\boldsymbol{q}_g),\ \boldsymbol{v}_0,\ r_0^{(g)}, L_0,\ (r_0'^{(g)} - L_0),\ r_1'^{(g)},\ L_0,\ L_1)\\
\boldsymbol{c}_1^{(g)} &\leftarrow \mathsf{SCROT}^{\dagger}(\mathsf{Enc}(g_0),\ \vec{\mathsf{Enc}}(\boldsymbol{q}_g),\ \boldsymbol{v}_1,\ r_1^{(g)}, L_1,\ r_0'^{(g)},\ r_1'^{(g)},\ L_0,\ L_1),\\
\boldsymbol{c'}_1^{(g)} &\leftarrow \mathsf{SCROT}^{\dagger}(\mathsf{Enc}(g_0),\ \vec{\mathsf{Enc}}(\boldsymbol{q}_g),\ \boldsymbol{v}_1,\ r_1^{(g)}, L_1,\ (r_0'^{(g)} - L_0),\ r_1'^{(g)},\ L_0,\ L_1),
\end{aligned}$$

$$\begin{aligned}
d_0 &\leftarrow \mathsf{isSCLongest}(\mathsf{Enc}(f_0 - r_0'^{(f)}),\ \mathsf{Enc}(g_0 - r_0'^{(g)}),\ \mathsf{Perm}(\vec{\mathsf{Enc}}(\boldsymbol{q}_f), r_1'^{(f)}), \mathsf{Perm}(\vec{\mathsf{Enc}}(\boldsymbol{q}_g), r_1'^{(g)}))\\
d_1 &\leftarrow \mathsf{isSCLongest}(\mathsf{Enc}(f_0 - r_0'^{(f)} + L_0),\ \mathsf{Enc}(g_0 - r_0'^{(g)}),\ \mathsf{Perm}(\vec{\mathsf{Enc}}(\boldsymbol{q}_f), r_1'^{(f)}), \mathsf{Perm}(\vec{\mathsf{Enc}}(\boldsymbol{q}_g), r_1'^{(g)}))\\
d_2 &\leftarrow \mathsf{isSCLongest}(\mathsf{Enc}(f_0 - r_0'^{(f)}),\ \mathsf{Enc}(g_0 - r_0'^{(g)} + L_0),\ \mathsf{Perm}(\vec{\mathsf{Enc}}(\boldsymbol{q}_f), r_1'^{(f)}), \mathsf{Perm}(\vec{\mathsf{Enc}}(\boldsymbol{q}_g), r_1'^{(g)}))
\end{aligned}$$

- Storing random values $r_0'^{(f)} \leftarrow r_0^{(f)},\ \ r_1'^{(f)} \leftarrow r_1^{(f)},\ \ r_0'^{(g)} \leftarrow r_0^{(g)},\ \ r_1'^{(g)} \leftarrow r_1^{(g)}$
- Sending $\boldsymbol{c}_0^{(f)}, \boldsymbol{c'}_0^{(f)}, \boldsymbol{c}_1^{(f)}, \boldsymbol{c'}_1^{(f)}, \boldsymbol{c}_0^{(g)}, \boldsymbol{c'}_0^{(g)}, \boldsymbol{c}_1^{(g)}, \boldsymbol{c'}_1^{(g)}, d$ to the user

(c) (*Decryption of the encrypted flag and the randomized interval*) The user performs the following steps:

if $(\mathsf{Dec}(d_0) == 0\ ||\ \mathsf{Dec}(d_1) == 0\ ||\ \mathsf{Dec}(d_2) == 0)$

Reports the result $S[1, \ldots, i-2]$ and sending the server decoy queries until $i == \ell$

else

Computes $f_0 \leftarrow \mathsf{SCChooseDec}(c_{0,f_0}^{(f)}, c'^{(f)}_{0,f_0}, L'_0)$, $g_0 \leftarrow \mathsf{SCChooseDec}(c_{0,g_0}^{(g)}, c'^{(g)}_{0,g_0}, L'_0)$,

$f_1 \leftarrow \mathsf{SCChooseDec}(c_{1,f_0}^{(f)}, c'^{(f)}_{1,f_0}, L_1)$, $g_1 \leftarrow \mathsf{SCChooseDec}(c_{1,g_0}^{(g)}, c'^{(g)}_{1,g_0}, L_1)$ ▷ for choosing correct results

end if

$i \leftarrow i + 1 \quad k \leftarrow k + 1$

end while

Algorithm S5 Building blocks for linear communication size $PBWT - sec$

function $\mathsf{isLongest}(\vec{\mathsf{Enc}}(\boldsymbol{q}_f),\ \vec{\mathsf{Enc}}(\boldsymbol{q}_g),\ r'^{(f)},\ r'^{(g)})$
  $\vec{\mathsf{Enc}}(\boldsymbol{q}'_f) = \mathsf{Perm}(\vec{\mathsf{Enc}}(\boldsymbol{q}_f), r'^{(f)})$
  $\vec{\mathsf{Enc}}(\boldsymbol{q}'_g) = \mathsf{Perm}(\vec{\mathsf{Enc}}(\boldsymbol{q}_g), r'^{(g)})$
  for $i = 0$ to M **do**
    Generating random value r
    $d = d \oplus \mathsf{Enc}\big(r \times (q'_f[i] - q'_g[i])\big)$
  end for
  return d
end function

function $\mathsf{isLongestGT}\epsilon(\vec{\mathsf{Enc}}(\boldsymbol{q}_f),\ \vec{\mathsf{Enc}}(\boldsymbol{q}_g),\ r'^{(f)},\ r'^{(g)},\ \epsilon)$
  for $k = 0$ to $\epsilon - 1$ **do**
    $\vec{\mathsf{Enc}}(\boldsymbol{q}'_f) = \mathsf{Perm}(\vec{\mathsf{Enc}}(\boldsymbol{q}_f), r'^{(f)})$
    $\vec{\mathsf{Enc}}(\boldsymbol{q}'_g) = \mathsf{Perm}(\vec{\mathsf{Enc}}(\boldsymbol{q}_g), r'^{(g)})$
    $\vec{\mathsf{Enc}}(\boldsymbol{q}'_g) = \mathsf{Perm}(\vec{\mathsf{Enc}}(\boldsymbol{q}_g), k)$ ▷ $\boldsymbol{q}'_f = \mathsf{Perm}(\boldsymbol{q}'_g, k)$ iff. $(g - f) = k$
    for $i = 0$ to M **do**
      Generating random value r
      $d = d \oplus \mathsf{Enc}\big(r \times (q'_f[i] - q'_g[i])\big)$
    end for
    $d_k = d$
  end for
  $\boldsymbol{d} = (d_0, \ldots, d_\epsilon)$
  Shuffling order of elements in $\boldsymbol{d}$
  return $\boldsymbol{d}$
end function
